# Knowledge and attitudes toward corneal donation among Singaporean youth: a cross-sectional study

**DOI:** 10.1186/s40662-016-0049-3

**Published:** 2016-07-04

**Authors:** Carisa Mariella Alvarez Paraz, Ha Thi Thu Truong, Darren Kyauk Sai, Howard Yu Cajucom-Uy, Cherry Lay Li Chan, Selina Md Kassim

**Affiliations:** Singapore Eye Bank, Singapore National Eye Centre, 11 Third Hospital Avenue, Singapore, 168751 Singapore; SingHealth Transplant, Singapore Health Services Pte Ltd, Transplant Administrative and Resource Office, Singapore General Hospital, Block 1, Level 3, 1 Hospital Drive, Singapore, 169608 Singapore; School of Applied Science, Republic Polytechnic 9 Woodlands Avenue 9, Singapore, 738964 Singapore

**Keywords:** Corneal donation, Donation knowledge, Willingness, Corneal transplant

## Abstract

**Background:**

To assess the knowledge of Singaporean youth regarding corneal donation and gauge their willingness to donate their corneas.

**Methods:**

We conducted a cross-sectional study among 500 students from five tertiary institutions in Singapore. All students answered self-administered questionnaires which included seven questions that tested knowledge and three questions that determined willingness to donate corneas.

**Results:**

Among 500 Singaporean youth aged 18 to 25, most students (73.2 %) answered 3 or fewer of the 7 questions about corneal donation correctly. With regards to the willingness to donate, 155 (31 %) were willing to donate their corneas, 111 (22.2 %) were not willing to donate their corneas, and 234 (46.8 %) were undecided. Willingness to donate corneas was associated with an older age group (21 to 25 years old), those who are non-Muslims, and have good basic knowledge. Particularly, students with good basic knowledge were 1.71 times more likely to willingly donate their corneas.

**Conclusion:**

The knowledge of the Singaporean youth regarding corneal donation and transplantation is poor. Since insufficient information was cited as the most common reason for being undecided in regards to corneal donation, specific and tailored programs to increase knowledge and awareness are needed to convince the youth to support corneal donation.

**Electronic supplementary material:**

The online version of this article (doi:10.1186/s40662-016-0049-3) contains supplementary material, which is available to authorized users.

## Background

Diseases affecting the cornea are the second most common cause of blindness worldwide, next to cataract [1, 2]. Fortunately, corneal blindness is fully reversible following a corneal transplant. Corneal transplantation is the most common type of transplant surgery, and have been performed since the first successful human corneal transplant in 1905 [3]. A successful corneal transplant begins with fresh and high quality corneas, ideally from local donors. However, the current corneal donor rate in Singapore is insufficient to meet the increasing demand for corneal transplants [4]. Unpublished data from the Singapore Eye Bank (SEB) shows that local corneas only make up a third of all corneas it received in 2014.

The reasons and factors behind a person’s willingness or lack thereof in donating their corneas after death are varied. An Australian study conducted in 2010 among 371 adults showed that the decision not to donate corneas, despite willingness to donate other organs, is due to concerns surrounding disfigurement [5]. A 2013 study among registered tissue donors in Nanjing, China, reported that well-educated males over 58 years of age with white-collar jobs and party affiliations (e.g., the Communist Party of China) tended to have more favourable views towards corneal donation [6]. In Singapore, a 2005 study showed that 67 % of adults surveyed were willing to donate their corneas, with ethnicity (Chinese) and religion (Christian, Hindu, and atheist) associated with increased willingness to donate corneas [7].

Among the studies on knowledge and perception of the youth on corneal donation, most of the studies we reviewed focused on medical and nursing students, which may give a skewed representation of knowledge among the youth [8–11]. In 2002, Dhaliwal reported that 79.6 % of surveyed medical students were well aware of corneal donation from deceased donors and 87.8 % were willing to donate corneas [11]. Gupta and colleagues found a high percentage of nursing students in Bangalore (85.1 %) who were willing to donate their eyes or were registered eye donors. These students also generally had good knowledge of the corneal donation process, with more than three quarter of them being able to answer two knowledge questions correctly [8]. Similarly, a study done in 2007 by Singh and colleagues found that 87.2 % of surveyed medical students reported their willingness to donate eyes in Delhi. However, less than half of those respondents were aware that eye donation should be done ideally within six hours of post death certification [9]. It was well known that campaigns for corneal and organ donation in recent years in India has been successful to a certain extent, especially in states like Tamil Nadu and Andhra Pradesh [12, 13]. In Africa, a survey among medical students of the University of Nigeria presented high awareness of corneal donation at 87 % but low donation willingness of 33.6 % [14]. A 2009 Malaysian study conducted on a more diverse group of university students showed 27 % of those surveyed were willing to donate their eyes, while the level of knowledge on corneal donation needed improvement [15]. All these studies were conducted in fairly homogenous cohorts of population in terms of race and religion. Singapore is a multiracial society with high tolerance for all religious practice. Thus, it was in our interest to understand the role of this social aspect in the willingness to donate corneas among the local population.

The youth have to be well-informed regarding corneal donation in order for them to make the appropriate choices regarding theirs and their relatives’ end-of-life decisions. The purpose of our study was to assess the knowledge of Singaporean youth regarding corneal donation and to gauge their willingness to donate their corneas. We chose youth as the target population of study for two main reasons. Firstly, characteristics of young people at this age make them desirable subjects for organ and tissue donation awareness (OTDA) campaign outreach. They are likely to be receptive to new ideas as they are still in an intensive learning process in their lives. Equipping the youth with correct facts about the current situation of low donation rate in Singapore and the related donation process will enable them to gain a positive attitude toward donation. Furthermore, OTDA education that appeals to their empathy, altruism and prosocial attitude at this stage of their life is more likely to make a campaign successful if carried out in the right way [16–18]. Secondly, it has been reported that OTDA efforts worked well in a youth-centred programme [19–22]. This study will provide a better understanding of their knowledge and attitudes, which will help formulate specific strategies to raise awareness and address concerns among this very important demographic. By reaching out specifically to this group and gaining acceptance for corneal donation, this may subsequently result in an increase in local donor rates.

## Methods

### Design

A questionnaire was given to those belonging to a specific age range (18 – 25 years old) to assess the knowledge of corneal donation and willingness to donate corneas. This age range was chosen following the definition of “youth” determined by the Singapore National Youth Council [23]. Under the Medical (Therapy, Research and Education) Act (MTERA), the legal age for one to pledge for organ and tissue donation in Singapore is 18 years old [24]. Thus, we chose this age as the lower age limit. This age restriction was also to ensure the age uniformity among survey populations of the five institutions, where the minimum entrance age is 17 years old for Polytechnics and 18 years old for Universities.

### Study population

We conducted a cross-sectional study using the convenience sampling method, with an equal number of students from the five tertiary institutions. The five tertiary institutions included the National University of Singapore, Nanyang Technological University, Ngee Ann Polytechnic, Republic Polytechnic, and Singapore Polytechnic. Participants were selected on a voluntary basis without any compensation given. All students answered self-administered questionnaires. A total of 600 students were approached with 559 students agreeing to participate in the study. After data collection, we excluded 59 questionnaires due to incompleteness (*n* = 43) or unmet inclusion criteria (*n* = 16). That left a total of 500 complete questionnaires for the study.

### The questionnaire

The survey form included demographic data (age, sex, race, nationality and religion) and 10 close-ended questions. The first seven questions tested knowledge and the last three questions determined willingness to donate corneas (Additional file 1). The questionnaire was validated for content validity by three study team members from the Singapore Eye Bank and two other researchers from the Singapore National Eye Centre. We pre-tested the questionnaire on five students and five data collectors who did not participate in the study and had no prior knowledge about the topic to ensure the questionnaire’s clarity and reliability [25, 26]. Data collection only commenced when the survey instrument had been confirmed of its readability, validity and clarity.

### Data collection and analysis

Trained survey data collectors approached students in the five local tertiary institutions and asked for consent to participate in the study. Verbal consent was given when students agreed to fill in the survey questionnaire. The data collectors distributed the questionnaires and remained on hand to answer questions from the study participants. They also made sure that the questionnaires were completely filled out to minimize any missing data. The database collection was approved by the ethics committee of the respective academic institutions where the study was executed.

In the knowledge section, all correct answers were given 1 point while incorrect answers were assigned a score of 0. Knowledge of participants was assessed based on their total score, which ranged between 0 and 7.

We analysed the data using IBM SPSS version 21, employing chi-square test or Fisher’s exact test for univariate analysis to determine an association between demographic factors and knowledge and attitudes towards corneal donation. We included all factors with an association of *p* value < 0.1 in multivariate modelling. Any test with *p* value < 0.05 was considered statistically significant.

## Results

Five hundred students from Singaporean universities and polytechnics participated in the survey. The mean age of study participants was 20.5 years (range: 18–25 years, standard deviation: 2.42), with 54.2 % aged 18–20 years old and 45.8 % aged 21–25 years old. Almost two-thirds of those surveyed were female students (63.6 %) while 36.4 % were male students. The majority of the participants were Singaporeans (85.8 %) while the rest were Permanent Residents (PR) and foreigners (5 % and 9.2 %, respectively). In terms of ethnicity, 62 % of those surveyed were Chinese, with Malay, Indian and Others (20.8 %, 12.4 % and 4.8 %, respectively) making up the rest of the participants. Religious affiliations are broken down as follows: Islam (25 %), Buddhism (24.6 %), Christianity (20.8 %), Hinduism (7.6 %), and Others (22 %).

The first seven questions in the survey tested the students’ knowledge regarding corneal donation and recovery, as summarized in Table 1. The mean for the number of correct answers is 2.76, with a standard deviation of 1.29. The percentage of participants who answered 3 or fewer questions correctly is 73.2 % while the percentage of participants who answered four or more questions correctly is 26.8 %.

**Table 1:** Knowledge of corneal donation and corneal recovery

Question	n (%)
Question 1: During corneal recovery, the part of the eye removed is	
The whole eyeball is removed. The corneas will be removed in the laboratory later on	18 (3.6)
The cornea which is the clear transparent window in front of the eye with the size of and shape of a contact lens (correct answer)	361 (72.2)
Don’t know	121 (24.2)
Question 2: The facial appearance will be altered after corneal recovery	
True	30 (6.0)
False (correct answer)	370 (74.0)
Don’t know	100 (20.0)
Question 3: Corneal donation can take place	
Only if the donor has perfect eyesight	27 (5.4)
Only if the donor does not have any eye infection at the point of donation (correct answer)	123 (24.6)
Only if the donor meets the age criteria	37 (7.4)
Only if the donor has no history of eye-related conditions or previous eye surgery	313 (62.6)
Question 4: Duration of corneal recovery is approximately	
30 min (correct answer)	39 (7.8)
2 h	75 (15.0)
4.5 h	54 (10.8)
Don’t know	332 (66.4)
Question 5: The individuals who will not benefit from corneal transplant surgery are	
People with poor vision due to cloudy cornea	29 (5.8)
People with scarred cornea	52 (10.4)
People with poor vision due to diabetes (correct answer)	230 (46.0)
Don’t know	189 (37.8)
Question 6: Corneal donation is covered under which legislation	
The Human Organ Transplant Act (HOTA)	273 (54.6)
The Medical (Therapy, Research and Education) Act	29 (5.8)
Neither	52 (10.4)
Both (correct answer)	146 (29.2)
Question 7: It is possible to specify who will receive the donated corneas	
True	177 (35.4)
False (correct answer)	110 (22.0)
Don’t know	213 (42.6)

The second part of the survey included three questions regarding willingness to donate corneas, as summarized in Table 2. A total of 155 participants (31 %) were willing to donate their corneas, 111 (22.2 %) were not willing to donate their corneas, and 234 (46.8 %) were undecided. The three most common reasons cited for being unwilling or undecided to donate corneas were: “I need more information about corneal donation and corneal transplantation”, “I think my family is not supportive of corneal donation”, and “I am worried of how my body will be treated after my death.” The three most common choices that would make the students feel more positive about corneal donation were: “If I understand corneal donation and corneal transplant process better”, “If I know a family member or a friend who needs corneal transplant to gain back his/her sight”, and “If I am assured that a donor’s body will be treated with full respect.”

**Table 2:** Willingness to donate corneas

Question	n (%)
Question 8: Are you willing to donate your corneas?	
Yes	155 (31.0)
No	111 (22.2)
Undecided	234 (46.8)
Question 9: If you are not willing or undecided to donate corneas, the reasons are: (multiple choices allowed)	
I need more information about corneal donation and corneal transplantation	222
I think my family is not supportive of corneal donation	72
I am worried of how my body will be treated after my death	68
I think my medical history may affect my eligibility to donate	35
My religion does not support corneal donation	20
Other reasons:	25
I am afraid of operation/pain	8
I will donate after I die	5
I simply do not want to	3
I have not thought about it	2
I want my eyes to see the world	2
Personal reason	2
I cannot make a decision	1
I am not sure about my religion’s view	1
Blind person can still be alive without corneal transplant	1
Question 10: Which of the following would make you feel more positive about corneal donation? (multiple choices allowed)	
If I understand corneal donation and corneal transplant process better	103
If I know a family member or a friend who needs corneal transplant to gain back his/her sight	87
If I am assured that a donor’s body will be treated with full respect	78
If I know my family is supportive of corneal donation	48
If I know for sure that my religion is supportive of corneal donation	15
Other reasons	11
Because when I don’t need any more, I should give it away	4
Helping another human being	3
If I know the corneal allocation system better	2
If I know and I am assured that it would be for a good cause	1
Personal reason	1

Table 3 shows the association between good basic knowledge of corneal donation and demographic factors. Good basic knowledge is defined as answering both questions 1 and 3 correctly, which were identified by the Eye Bank staff as the most crucial items to know among the questions. Both univariate and multivariate analysis found the female gender to be associated with good basic knowledge. In the multivariate analysis, female participants were 1.89 times more likely than males to answer both questions correctly (95 % CI 1.09-3.29, *p* = 0.02) after adjusting for institution and nationality.

**Table 3:** Association between good basic knowledge of corneal donation (answered both questions 1 & 3 correctly) and demographic factors

Factor (% with good knowledge)	Unadjusted		Adjusted	
	OR (95 % CI)	*p* value	OR (95 % CI)	*p* value
Gender				
Male (12.1 %)	1^a^		1	
Female (19.5 %)	1.61 (1.03–2.53)	0.035	1.89 (1.09–3.29)	0.02
Age				
18–20 years old (16.2 %)	0.93 (0.63–1.37)			
21–25 years old (17.5 %)	1	0.72	-	
Nationality				
Singaporean (17.0 %)	0.74 (0.35–1.55)			
Permanent Resident (4.0 %)	0.15 (0.02–1.25)			
Foreigner (21.7 %)	1	0.21	-	
Race				
Chinese (18.4 %)	2.48 (0.57–10.8)			
Malay (11.5 %)	1.43 (0.30–6.89)			
Indian (21.0 %)	2.92 (0.01–14.0)			
Others (8.3 %)	1	0.22	-	
Religion				
Christianity (12.5 %)	0.57 (0.27–1.20)			
Buddhism (20.3 %)	1.02 (0.54–1.94)			
Islam (13.6 %)	0.63 (0.31–1.26)			
Hinduism (18.45)	0.90 (0.35–2.32)			
Others (20.0 %)	1	0.38	-	
Institution				
National University of Singapore (25.0 %)	1.63 (0.82–3.25)		1.59 (0.79–3.21)	
Nanyang Technological University (16.0 %)	0.93 (0.44–1.96)		0.92 (0.43–1.95)	
Singapore Polytechnic (10.0 %)	0.54 (0.24–1.25)		0.54 (0.23–1.26)	
Republic Polytechnic (16.0 %)	0.93 (0.44–1.96)		0.73 (0.34–1.58)	
Ngee Ann Polytechnic (17.0 %)	1	0.09	1	0.07

^a^1: Reference group

Table 4 shows the association between willingness to donate corneas and demographic factors. In binary regression modelling, three factors were found to be associated with willingness to donate corneas: older age group, non-Muslims and good basic knowledge. The older group (21 to 25 years old) was 2.46 times more likely to be willing to donate corneas than the younger group (95 % CI: 1.55-3.88, *p* < 0.001) after adjusting for race, institution, knowledge score and religion. Participants from all other religions were 3.65 times more likely to report their willingness to donate corneas compared to their Muslim counterparts (95 % CI: 1.09-12.22, *p* = 0.04) after adjustment for the rest of the factors. Students with good basic knowledge are 1.71 times more likely to report their willingness to donate corneas (95 % CI: 1.03-2.84, *p* = 0.04) after adjusting for other factors.

**Table 4:** Association between willingness to donate corneas (Yes) and demographic factors

Factor (% willing)	Unadjusted		Adjusted	
	OR (95 % CI)	*p* value	OR (95 % CI)	*p* value
Gender				
Male (29.1 %)	0.91 (0.69–1.19)			
Female (32.1 %)	1^a^	0.55	-	
Age				
18–20 years old (22.5 %)	1		1	
21–25 years old (41.0 %)	1.82 (1.39–2.39)	<0.001	2.46 (1.55–3.88)	<0.001
Nationality				
Singaporean (30.3 %)	0.62 (0.33–1.15)			
Permanent Resident (24.0 %)	0.45 (0.15–1.33)			
Foreigner (41.3 %)	1	0.23	-	
Race				
Chinese (33.9 %)	0.87 (0.49–1.53)		0.81 (0.44–1.50)	
Malay (20.2 %)	0.43 (0.21–0.87)		1.36 (0.38–4.83)	
Indian (37.1 %)	1		1	
Others (25.0 %)	0.56 (0.19–1.63)	0.04	0.81 (0.27–2.43)	0.83
Religion (Islam vs others)				
Islam (19.2 %)	1		1	
Other religions (34.9 %)	1.82 (1.23–2.67)	0.001	3.65 (1.09–12.22)	0.04
Institution				
National University of Singapore (41.0 %)	1.79 (0.99–3.23)		0.93 (0.48–1.79)	
Nanyang Technological University (37.0 %)	1.51 (0.83–2.74)		0.90 (0.46–1.75)	
Singapore Polytechnic (22.0 %)	0.73 (0.38–1.38)		0.74 (0.38–1.43)	
Republic Polytechnic (27.0 %)	0.95 (0.51–1.77)		0.92 (0.47–1.77)	
Ngee Ann Polytechnic (28.0 %)	1	0.02	1	0.93
Good basic knowledge				
Yes (41.7 %)	1.44 (1.07–1.94)		1.71 (1.03–2.84)	
No (28.8 %)	1	0.027	1	0.04

^a^1: Reference group

Table 5 shows the association between ambiguity to donate corneas (undecided) and demographic factors. Only age was found to be associated with ambiguous willingness to donate corneas in multi-variate analysis. The younger group (below 21 years old) was 2.07 times more likely than the older group to report that they are undecided when it comes to donating their corneas (95 % CI: 1.36-3.14, *p* = 0.001) after adjusting for gender, institution, and basic knowledge score. The reasons associated with ambiguity to donate corneas include: needing more information about corneal donation and transplantation, thinking the family is not supportive of corneal donation, concerns about how the body will be treated after death and thinking that the medical history will affect eligibility to donate (Table 6).

**Table 5:** Association between ambiguity to donate corneas (Undecided) and demographic factors

Factor (% undecided)	Unadjusted		Adjusted	
	OR (95 % CI)	*p* value	OR (95 % CI)	*p* value
Gender				
Male (51.6 %)	1.17 (0.97–1.41)		1.41 (0.94–2.12)	
Female (44.0 %)	1^a^	0.113	1	0.09
Age				
18–20 years old (55.0 %)	1.48 (1.21–1.81)		2.07 (1.36–3.14)	
21–25 years old (37.1 %)	1	<0.001	1	0.001
Nationality				
Singaporean (45.7 %)	0.92 (0.50–1.69)		-	
Permanent Resident (64.0 %)	1.94 (0.71–5.28)			
Foreigner (41.8 %)	1	0.21		
Race				
Chinese (46.5 %)	0.99 (0.57–1.71)		-	
Malay (48.1 %)	1.05 (0.56–1.98)			
Indian (46.8 %)	1			
Others (45.8 %)	0.96 (0.37–2.48)	0.99		
Religion				
Christianity (43.3 %)	1.14 (0.66–1.97)		-	
Buddhism (53.7 %)	1.74 (1.03–2.92)			
Islam (48.8 %)	1.43 (0.85–2.40)			
Hinduism (47.4 %)	2.35 (0.6–2.84)			
Others (40.0 %)	1	0.21		
Institution				
National University of Singapore (41.0 %)	0.69 (0.39–1.21)		1.04 (0.56–1.91)	
Nanyang Technological University (41.0 %)	0.69 (0.39–1.21)		1.04 (0.56–1.92)	
Singapore Polytechnic (59.0 %)	1.44 (0.82–2.51)		1.43 (0.81–2.52)	
Republic Polytechnic (43.0 %)	0.75 (0.43–1.32)		0.98 (0.54–1.78)	
Ngee Ann Polytechnic (50.0 %)	1	0.05	1	0.71
Good basic knowledge				
Yes (38.1 %)	1		1	
No (48.6 %)	1.27 (0.95–1.70)	0.093	1.43 (0.87–2.34)	0.16

^a^1: Reference group

**Table 6:** Association between ambiguity to donate corneas (undecided) and reasons

Reason (% undecided)	Unadjusted OR (95 % CI)	*p* value
I need more information about corneal donation and corneal transplantation		
Yes (80.1 %)	3.92 (3.08–4.97)	
No (20.4 %)	1^a^	<0.001
I think my family is not supportive of corneal donation		
Yes (58.3 %)	1.30 (1.04–1.62)	
No (44.9 %)	1	0.041
I am worried of how my body will be treated after my death		
Yes (63.25)	1.43 (1.16–1.76)	
No (44.2 %)	1	0.004
I think my medical history may affect my eligibility to donate		
Yes (81.3 %)	1.83 (1.50–2.22)	
No (44.4 %)	1	<0.001
My religion does not support corneal donation		
Yes (35.0 %)	1	
No (47.3 %)	1.35 (0.74–2.47)	0.36
Other reasons:		
Yes (44.8 %)	1	
No (46.9 %)	1.05 (0.69–1.58)	0.85

^a^1: Reference group

## Discussion

In our study, only 31 % of the population (students aged 18–25) were willing to donate their corneas compared to 67 % of Singaporean adults (aged 21–65) [7]. However, the older age bracket in our study (21–25 years old) was 2.46 times more likely to be willing to donate their corneas compared to the younger bracket (18–20 years old). We could probably attribute this to the organ and tissue donation booklet that the Ministry of Health (MOH) sends to all Singaporeans and PRs when they turn 21 [27]. The booklet, “Understanding HOTA,” contains information on the laws governing organ donation in Singapore and their implications on Singaporean citizens and PRs. Good basic knowledge on corneal donation is associated with willingness to donate, which may be why the age group assumed to have received the booklet recently is more willing to donate.

Another factor associated with willingness to donate is religion. In our study, all other religions were 3.65 times more likely to donate corneas compared with Islam. Similarly, among Singaporean adults, those who practiced Islam were the least willing to donate their corneas [7]. Gatrad cited in 1994 that organ transplantation has not been discussed in depth in the Koran, with different beliefs between Muslims of different countries [28]. Since tissue and organ donation and transplantation are not explicitly mentioned in the Koran, it is possible that other Muslim customs surrounding death affect the youth’s perception on becoming donors. For example, the Muslims require that the burial should be performed as soon as possible after death and that the body is kept whole [28, 29].

When compared to their international counterparts, Singaporean youth were slightly more willing to donate their corneas than Malaysian youth (31 % vs 27 %), Nigerian youth (14.3 %) but less so compared to Iranian youth (67.8 %) [15, 30, 31]. All the respondents in the Malaysian, Iranian and Nigerian studies were more homogenous in age, versus the wider 18–25 age bracket in the Singaporean study. It would have been interesting to compare willingness among different age brackets. Likewise, there was no mention of the students’ religions in the other three studies. Since age and religion were associated with willingness to donate in our study, having this data from these studies would have aided in making comparisons. The Malaysian students had fairly poor knowledge of eye donation, and this may have contributed to them being less willing to donate. It is important to point out that the Malaysian study focused on whole eye donation while the Singaporean studies (both in adults and the youth) focused on corneal donation. In our experience, people generally tend to be more accepting of cornea donation compared with whole eye donation, which is perceived to be disfiguring.

The most common reason for being undecided on corneal donation among Singaporean youth is the lack of information about corneal donation and transplantation. Providing adequate and accurate information about corneal donation and transplantation may in fact be the key to convince this demographic to support corneal donation. In contrast, Singaporean adults tend to cite the importance of the leaving the body intact after death as the most important reason for not donating corneas. Similarly, a study that focused on a group that was willing to donate organs and tissues but were specifically not willing to donate their eyes cited disfigurement as the main reason for not being willing to donate [5]. The study on Malaysian students did not identify the main reason for unwillingness to donate [15].

Overall, the knowledge of the Singaporean youth regarding corneal donation and transplantation is poor, with only 26.8 % answering at least 4 out of 7 questions correctly. However, 72 % of respondents correctly answered that only the cornea is removed during the procedure, whereas only 40 % of Malaysian students were aware that the current practice of eye donation in their country is whole eye removal [15]. Seventy-four percent (74 %) of Singaporean youth correctly answered that there is no disfigurement after corneal recovery. This is probably the reason why disfigurement does not rank high among the reasons for unwillingness to donate. A small number of respondents chose not to donate, citing the reason that they needed their eyes to see while they are still alive (Table 3). That reflected another misunderstanding that local corneal donation practice can be done through a live donor. This misconception also exists among Malaysian students, of whom 55.25 % believe that the eye can be removed from a living person for donation [15].

It should be noted that the vast majority of corneas recovered by the SEB are through cornea in situ excision rather than enucleation. The reasons for that are (1) legislative constrain and (2) best practice guideline. HOTA only allows removal of the corneas and not whole eyes. Under MTERA, the pledger can donate a whole eye for therapy and research but there has been very few cases of such request [27]. In terms of practice, cornea in situ recovery is well accepted and the preferred mode of cornea retrieval in the eye banking community [32–34]. Thus in Singapore, donated corneas are mainly recovered using in situ corneoscleral disc excision. This restriction does have an impact on the availability of human eye tissue for sight-saving research as Williams and colleagues summarized in their Viewpoint in 2016 [35]. Until Singapore can provide a sufficient number of corneas to meet the local transplant demand, it might be a long way to have adequate human eye tissues banked for research purposes.

Since lack of information on corneal donation and transplantation is cited as the main reason why Singaporean youth are hesitant to donate, increasing this age group’s knowledge may be the key in increasing the number of people willing to become donors. Aside from mailing the booklets from MOH to all 21 year olds, more interactive methods can be employed such as conducting lectures among university and polytechnic students, roadshows, and projects that would make the youth more aware about the two legislation Acts governing organ and tissue donation in Singapore. The SEB has been conducting facility tours and lectures about eye donation and eye banking for local tertiary students. However, this was only done on ad-hoc basis when requested from the institutions. We hope that more systematic methods to communicate to youths about this topic will be allowed in the future. One example would be having a short lecture about organ and tissue donation integrated into the tertiary education curriculum. This method was reported to be effective among medical students in the US and Germany [36, 37]. Taking a further step was the Gerundium program in Hungary. Medical students were trained to give lectures to high schools and colleges about organ and tissue donation [38]. We will need to engage with medical school educators to initiate this. Another approach to raise the youth’s awareness is through online outreach. With an increasing number of youths using social media, leveraging this platform may also be a way to get the message across. One example of such an initiative was campaigns by Stefanone and colleagues. Three online formats of Facebook ads, Student Seeders’ Social Networking Sites campaigns and Challenge Campaigns were used. Online advertisings showed wide outreach but the involvement of student seeders and challenge teams was an essential catalyst to behaviour change [39]. Collaboration between Donate Life America, Johns Hopkins University and Facebook saw approximately 33,000 Facebook members choosing the Facebook Organ Donor option. In the two weeks of the initiative, the mass effect was evident. However, the authors proposed using online tools as part of a larger campaign rather than single medium approach [40]. In Singapore, our future campaigns can consider interactive activities on popular social media websites and mobile apps to engage youths more actively in promoting corneal donation awareness. It is worth noting that the impact of campaign on youths will only be assessed at a much later time due to a decades-long lag time between time of pledging and time of expiration and organ recovery.

Despite a small sample size, this pilot study provided an important finding: youth’s reasons for low donation rates. We would like to expand the study further in the future, looking at their preferred mode for information dissemination as well as identifying the best means for engaging them in donor awareness activities. We are also interested to learn the efficacy of the HOTA booklet in imparting relevant donor related information to the youth.

## Conclusion

Among Singaporean youths aged 18 to 25, 31 % were willing to donate their corneas, and majority had poor knowledge on corneal donation and transplantation. The most common reason for being undecided to donate corneas was participants’ insufficient knowledge of corneal donation and transplantation. We need to work with the Ministry of Health, related government agencies, and local tertiary institutions to deliver specific and tailored programs to increase the youth’s knowledge and awareness of corneal donation and thereby increase local donor rates.

## Additional file

Additional file 1: Questionnaire items. (DOC 107 kb)
