# Supplementary material for: Knowledge and attitudes toward corneal donation among Singaporean youth: a cross-sectional study
Source: Eye Vis (Lond). 2016 Jul 4;3:17. doi: 10.1186/s40662-016-0049-3 (PMC4931698; doi:10.1186/s40662-016-0049-3)
Supplement: Additional file 1: — Questionnaire items. (DOC 107 kb) [file 40662_2016_49_MOESM1_ESM.doc]

| Additional file 1: Questionnaire items |
| --- |
| *Knowledge about corneal donation and recovery*   1. During corneal recovery, that part of the eye removed is: (choose one option only) 2. The whole eye ball is removed. The corneas will be removed in the laboratory later on 3. The cornea which is the clear transparent window in front of the eye with the size of and shape of a contact lens 4. Don’t know 5. The facial appearance will be altered after corneal recovery (choose one option only)   a. True b. False c. Don’t know   1. Corneal donation can take place (choose one option only)   a. Only if the donor has perfect eyesight  b. Only if the donor does not have any eye infection at the point of donation  c. Only if the donor meets the age criteria  D. Only if the donor has no history of eye-related conditions or previous eye surgery   1. Duration of corneal recovery is approximately 2. 30 minutes 3. 2 hours 4. 4.5 hours 5. Don’t know 6. The individuals who will not benefit from corneal transplant surgery are 7. People with poor vision due to cloudy cornea 8. People with scarred cornea 9. People with poor vision due to diabetes 10. Don’t know 11. Corneal donation is covered under which legislation: 12. The Human Organ Transplant Act (HOTA) 13. The Medical (Therapy, Research and Education) Act 14. Neither 15. Both 16. It is possible to specify who will receive the donated corneas 17. True b. False c. Don’t know |
| *Willingness to donate corneas*   1. Are you willing to donate your corneas? 2. Yes (please proceed to question 10) 3. No (please proceed to question 9) 4. Undecided (please proceed to question 9) 5. If you are NOT willing or undecided to donate corneas, the reasons are: (you may choose more than one answer) 6. My religion does not support corneal donation 7. I think my family is not supportive of corneal donation 8. I am worried of how my body will be treated after my death 9. I think my medical history may affect my eligibility to donate 10. I need more information about corneal donation and corneal transplantation 11. Other, specify: _____________________________________________________________________ 12. Which of the following would make you feel more positive about corneal donation? (you may choose more than one answer) 13. If I understand corneal donation and corneal transplant process better 14. If I am assured that a donor’s body will be treated with full respect 15. If I know my family is supportive of corneal donation 16. If I know for sure that my religion is supportive of corneal donation 17. If I know a family member or a friend who needs corneal transplant to gain back his/her sight 18. Other, specify: ____________________________________________________________________ |
